# Lupus and IgA nephropathy: coexistence or coincident?

**DOI:** 10.1007/s00508-025-02512-y

**Published:** 2025-03-19

**Authors:** Rahime Duran, Derya Yıldırım, Rıza Can Kardaş, İbrahim Vasi, Burcugül Kaya, Yahya Çakır, İbrahim Karaduman, Hamit Küçük, Berna Göker, Mehmet Akif Öztürk, Abdulsamet Erden

**Affiliations:** Division of Rheumatology, Department of Internal Medicine, Gazi University Faculty of Medicine, Ankara, Turkey

**Keywords:** IgA nephropathy, Prognosis, Kidney failure, Lupus nephritis, Systemic lupus erythematosus

## Abstract

**Background:**

Systemic lupus erythematosus is a multisystem autoimmune disease primarily manifesting as lupus nephritis. While lupus nephritis is the most common renal pathology in lupus, non-lupus nephropathies such as IgA nephropathy occasionally occur. This study aims to evaluate the clinical features and outcomes of lupus patients with IgA nephropathy, comparing them with those of primary IgA nephropathy and lupus nephritis.

**Methods:**

A comprehensive literature review was conducted using the PubMed and Google Scholar databases to identify cases of systemic lupus erythematosus with IgA nephropathy reported between 1995 and December 2023. A total of 16 cases were identified and 2 additional cases from our clinic were included. These cases were compared with 47 lupus nephritis patients from our clinic and 215 primary IgA nephropathy patients from the literature. Data were collected on demographics, serology, renal biopsy findings, treatment, progression to renal failure and mortality.

**Results:**

We identified 18 cases of lupus with IgA nephropathy with a median age of 41.6 years and a female predominance (72.2%). In comparison to the primary IgA nephropathy cohort, lupus with IgA nephropathy group exhibited a lower rate of renal failure (11.1% vs. 34%) and mortality (5.6% vs. 20%). Additionally, the lupus-IgA nephropathy group showed a slightly lower mortality rate compared to the lupus nephritis cohort (5.6% vs. 10.6%).

**Conclusion:**

Lupus with predominantly IgA deposits often follows a more indolent course than primary IgA nephropathy but severe cases with crescentic glomerulonephritis can still progress to renal failure.

## Introduction

Systemic lupus erythematosus (SLE) is a multisystem autoimmune disease, with approximately 50% of patients developing renal involvement during the disease course. Lupus nephritis (LN) can be categorized into six subtypes: minimal mesangial (class I), mesangial proliferative (class II), focal proliferative (class III), diffuse proliferative (class IV), membranous nephropathy (class V) and advanced sclerosing (class VI). Typical LN is characterized by a “full house” pattern under immunofluorescent microscopy, with positive staining for IgG, IgA, IgM, C3 and C1q [1]. In addition to these subtypes, non-lupus nephropathies in SLE patients have rarely been reported. One such nephropathy is IgA nephropathy (IgAN), which is the most common primary glomerulonephritis worldwide [2].

The co-occurrence of IgAN with other autoimmune diseases is not very common; however, some studies have suggested that it may be particularly associated with diseases such as SLE, rheumatoid arthritis, inflammatory bowel disease, celiac disease and ankylosing spondylitis. Although the coexistence of IgAN in SLE is rare and underexplored, it has important implications for treatment and prognosis [3].

In this study, we reviewed the literature regarding SLE patients with IgAN. We also evaluated similar patients from our clinic and described their clinical features. Furthermore, we compared these patients with the LN cohort from our clinic and the primary IgAN cohort from the literature. Our aim was to evaluate the clinical features and outcomes of SLE patients with IgAN, comparing them with those of primary IgAN and SLE patients with LN.

## Methods

### Study population

We conducted a case-based review of the PubMed and Google Scholar databases using MeSH keywords “systemic lupus erythematosus” and “IgA nephropathy” to identify previously reported cases involving SLE patients with IgAN published from 1995 to December 2023. Publications in the English language were included. To be eligible for review, articles of any type (original articles, case series, and case reports) reporting on individual SLE patients with IgAN were selected. Articles not including individual patient characteristics were excluded. The initial selection process was based on titles and abstracts of retrieved articles, followed by a full-text review of relevant articles. Ultimately, 11 publications reporting related cases were included in our literature review, covering a total of 16 patients. Additionally, we included 2 other cases with similar properties that were followed in our clinic. All 18 cases met the 1982 or 2019 American College of Rheumatology diagnostic criteria for SLE [4, 5]. The diagnosis of IgAN in all cases was based on findings from light microscopy, immunofluorescence (predominant IgA deposition in the mesangium) and electron microscopy of kidney biopsy specimens.

We selected a recently published cohort of 215 patients with biopsy-proven primary IgAN as the control group, documenting median age, gender, serum creatinine (mg/dl), proteinuria (g/g creatinine), end-stage kidney disease (ESKD) rate and mortality rate [6]. We also described similar parameters of 47 LN patients (classes III, IV, or III/IV + V) out of a total of 517 SLE patients under follow-up in our rheumatology clinic.

### Study design

Data regarding baseline demographics, comorbidities, antinuclear antibody (ANA), anti-dsDNA, complement C3 and C4 levels, serum creatinine, 24‑h urine protein, hematuria (microscopic or gross), renal biopsy findings, pharmacotherapy, progression to ESKD and mortality were documented in Table 1 for all cases. As anti-dsDNA titers, C3, C4 and serum creatinine levels were not consistently documented numerically for all cases and reference ranges can vary across studies, we defined the anti-dsDNA titer as positive or negative, C3/C4 levels as normal or low, and serum creatinine levels as normal or elevated by evaluating each study based on its own values. In some studies, post-treatment proteinuria levels were reported quantitatively, while in others, they were described as a good response to treatment.

**Table 1:** Summary of characteristics of 18 patients of SLE with IgA nephropathy

First author and year	Age/sex	ANA	Anti-dsDNA	C3/C4	Creatinine	Proteinuria	Hematuria	Comorbidity	Renal biopsy	Treatment	Prognosis
Lai (1995) [7]	19/F	1/1280	Positive	Low	Normal	1.5 g/24h	Gross	–	Modest segmental mesangial hypercellularity	NA	FUO-exitus
	34/F	1/320	Negative	Normal	Normal	5.3 g/24h	Gross	HT	Modest segmental mesangial hypercellularity, segmental sclerosis	5–10 mg prednisone/day	Proteinuria: 2 g/24h
	28/F	1/640	Negative	Normal	Normal	1.8 g/24h	Microscopic	–	Modest segmental mesangial hypercellularity	10 mg prednisone/day	Proteinuria: 1.1 g/24h
Basile C (1998) [8]	25/M	1/640	Positive	Normal	Normal	1 g/24h	Microscopic	Hashimoto	Modest segmental mesangial hypercellularity	1 mg/kg day^−1^ prednisone	Proteinuria: 1 g/24h persisted
Corrado (2007) [9]	43/F	1/180	Positive	Low	Normal	NA	Microscopic	HT	Slight mesangial proliferation, sclerosis, crescents	Low dose prednisone, ACE‑inhibitor	Good response
Horino (2010) [10]	22/M	1/2560	Positive	Normal	Normal	1.8 g/24h	Microscopic	–	1st renal biopsy: class IIB; 2nd biopsy: moderate mesangial proliferation + crescents	Prednisone 1 mg/kg day^−1^ for 1 month then tapered; after re-biopsy: 50 mg losartan, 300 mg dipyridamole	Proteinuria: 100 mg/24h
Kobak (2011) [11]	46/F	1/640	Positive	Low	Normal	500 mg/24h	Microscopic	Hashimoto	Mesangial hypercellularity, crescents	1 mg/kg day^−1^ prednisone, chloroquine 250 mg/day, ACE-inhibitor 5 mg/day	Good response
Hongyan (2013) [12]	64/F	1/320	Negative	Low	Normal	2393 mg/24h	Gross	–	Mild diffuse mesangial hyperplasia	1 mg/kg day^−1^ prednisone + IV CYC	Good response
	45/M	1/320	Positive	Low	Elevated	2497 mg/24h	Gross	–	Mild diffuse mesangial hyperplasia, glomerular sclerosis	1 mg/kg day^−1^ prednisone + IV CYC	Good response
	31/F	1/320	Positive	Normal	Normal	651 mg/24h	Microscopic	–	Mild diffuse mesangial hyperplasia	1 mg/kg day^−1^ prednisone	Good response
	31/F	1/3200	Negative	Low	Elevated	3619 mg/24h	Microscopic	–	Mild diffuse mesangial hyperplasia	1 mg/kg day^−1^ prednisone	Good response
	40/M	1/3200	Positive	Low	Normal	4692 mg/24h	Microscopic	–	Mild diffuse mesangial hyperplasia	1 mg/kg day^−1^ prednisone + IV CYC	Good response
Silva (2015) [13]	40/F	1/1280	Negative	Low	Normal	1045 mg/24h	Microscopic	–	Mesangial hypercellularity	60 mg/day prednisone, hydroxychloroquine 400 mg/day, enalapril 10 mg/day	Good response
Patel (2019) [14]	74/F	1/320	Positive	Normal	Elevated	1480 mg/24h	Microscopic	HT, DM, HF, CKD	Mesangial proliferative glomerulonephritis with crescents	500 mg MPZ for 3 days then 60 mg prednisone + 5× plasmapheresis	ESKD
Jiang (2023) [15]	72/M	1/100	Negative	Low	Elevated	NA	Microscopic	HT	Mesangial hypercellularity with crescents	500 mg MPZ for 3 days then 60 mg prednisone + CYC	ESKD
Cuello (2023) [16]	64/F	1/320	Positive	Normal	Normal	82 mg/24h	Microscopic	–	Mild mesangial hypercellularity	Prednisone, ACE-inhibitor	Good response
Our case #1	41/F	1/160	Negative	Low	Normal	4.3 g/24h	Microscopic	HT	Mesangial hypercellularity, glomerulosclerosis	Prednisone, ACE-inhibitor	Proteinuria: 1 g/24h
Our case #2	31/F	1/320	Negative	Normal	Normal	1.6 g/24h	Microscopic	–	Mesangial hypercellularity	60 mg prednisone, irbesartan 1 × 150 mg	Proteinuria: 150 mg/24h

*ACE* angiotensin-converting enzyme, *ANA* anti-nuclear antibody, *anti-dsDNA* anti-double-stranded DNA, *C3* complement factor 3, *C4* complement factor 4, *CKD* chronic kidney disease, *CYC* cyclophosphamide, *DM* diabetes mellitus, *ESKD* end stage kidney disease, *F* female, *FOU* fever of unknown origin, *HF* heart failure, *HT* hypertension, *M* male, *MPZ* methylprednisolone, *NA* not available, *PLEX* plasma exchange, *SLE* systemic lupus erythematosus

### Statistical analysis

Statistical analyses were conducted to compare demographic and clinical characteristics among the patient groups: SLE with IgA nephropathy (SLE-IgAN), primary IgA nephropathy (primary IgAN), and SLE with lupus nephritis (SLE-LN). Age and proteinuria levels, as non-parametric data, were analyzed using the Kruskal-Wallis test followed by Dunn-Bonferroni post hoc analyses. Categorical variables including gender, anti-dsDNA positivity, decreased complement levels, use of pharmacotherapy (prednisone, cyclophosphamide, angiotensin-converting enzyme, ACE, inhibitor), incidence of end-stage kidney disease (ESKD), and mortality were compared using the χ^2^-test. For pairwise comparisons of categorical variables, Fisher’s exact test was employed with *p*-values adjusted using the Holm-Bonferroni method. Statistical analysis was performed using SPSS (version 26, IBM, Armonk, NY, USA) with significance set at *p* < 0.05.

## Results

### Clinical and demographic features

We identified 18 cases of SLE with IgA nephropathy, as detailed in Table 1. The median age at diagnosis was 41.6 years (range 19–74 years) for SLE patients with IgA deposits. Of the 18 patients 13 were female. The primary clinical presentation included persistent microscopic hematuria and varying levels of proteinuria in 15 patients, while 3 patients presented with gross hematuria. Anti-dsDNA titers were elevated in 10 patients and negative in 8 patients. Complement levels were low in 10 patients and normal in 8 patients. Of the patients 4 exhibited nephrotic range proteinuria (> 3.5 g/24 h), with a median proteinuria level recorded at 2141 mg/24 h (range 82–5300 mg/24 h). Acute kidney injury was observed in two patients. Biopsy of the kidneys revealed predominant deposition of IgA in the mesangium in all patients, with crescent formation observed in 4 patients. Additionally, a 22-year-old male patient previously diagnosed with class II LN underwent a second renal biopsy due to increased proteinuria without any clinical or immunological activity related to SLE. The second biopsy confirmed the presence of IgA nephropathy.

In the primary IgAN cohort, the median age of patients was 44 years, with 71% being male. Renal function tests revealed a median proteinuria of 1.24 g/g creatinine (range 0.62–2.36 g/g creatinine) and serum creatinine of 1.79 mg/dl (range 1.30–2.74mg/dl).

In the SLE-LN cohort the median age of patients was 30 years, with 90.9% being female. Anti-dsDNA positivity was found in 74.5% of patients and decreased complement levels in 76.6% of patients. Detailed characteristics of the patients are documented in Table 2.

**Table 2:** Characteristics and clinical features of patients with SLE-IgAN, primary IgAN and SLE-LN

	SLE-IgAN (*n* = 18)	Primary IgAN (*n* = 215)	SLE-LN (*n* = 47)	*P*
*Age*, median (range), years	41.6 (19–74)	44 (34–58)	30 (18–55)	*<* *0.001*
*Gender, n* (%)				
Female	13 (72.2)	63 (29)	40 (90.9)	*< 0.001*
Male	5 (27.7)	152 (71)	4 (9.1)	
*Hematuria, n* (%)				
Microscopic	15 (83.3)	N/A	26 (68.4)^1^	*<* *0.001*
Gross	3 (16.6)	N/A	–	
*Proteinuria*, median (range), mg/24 h	2141 (82–5300)	1240 (620–2360)	2141 (293–10,883)	*<* *0.001*
*Anti-dsDNA positivity, n* (%)	10 (55.5)	N/A	35 (74.5)	*<* *0.001*
*Decreased complement *(C3 or C4), *n* (%)	10 (55.5)	N/A	36 (76.6)	*<* *0.001*
*Medical treatment, n* (%)				
Prednisone	17 (94.4)	N/A	47 (100)	0.304
CYC	4 (22.2)	92 (43)	28 (55.3)	*0.017*
ACE‑inhibitors	7 (38.8)	133 (62)	34 (72.3)	*0.044*
*ESKD, n* (%)	2 (11.1)	73 (34)	5 (10.6)	*<* *0.001*
*Mortality, n* (%)	1 (5.6)	43 (20)	5 (10.6)	*0.120*

*ACE* Angiotensin-converting enzyme, *anti-dsDNA* anti-double-stranded DNA, *CYC* Cyclophosphamide, *ESKD* end stage kidney disease, *IgAN* IgA nephropathy, *SLE* systemic lupus erythematosus

^1^ Hematuria for SLE with LN group is not stratified into gross and microscopic

### Treatment and follow-up

Most patients received glucocorticoids in the SLE-IgAN group. Additionally, four patients required intravenous cyclophosphamide (CYC) as an immunosuppressive agent. In one case of rapidly progressive glomerulonephritis, plasmapheresis was administered. During follow-up, most patients demonstrated a favorable prognosis; however, several complex and refractory cases were also noted. For instance, a 72-year-old man presented with acute progressive glomerulonephritis and received high-dose pulse glucocorticoid treatment along with cyclophosphamide pulse treatment but the renal function failed to recover, necessitating continued hemodialysis. Similarly, a 74-year-old woman, known to have SLE and chronic kidney disease attributed to diabetes mellitus and hypertension, experienced acute kidney injury. Despite receiving pulse methylprednisolone and plasmapheresis, the renal function did not recover, prompting initiation of hemodialysis. A 19-year-old female patient with cerebral lupus, died due to an unknown systemic infection. In the primary IgA nephropathy group, 34% of 215 patients progressed to ESKD with a reported mortality rate of 20% [6].

In the SLE-LN cohort, 42 patients (89.3%) received mycophenolate mofetil, 28 patients (59.5%) received azathioprine, 26 patients (55.3%) received calcineurin inhibitors and 6 patients (12.8%) were treated with rituximab. Of the 47 patients 10.6% progressed to ESKD, with a reported mortality rate of 10.6%. Detailed patient characteristics are provided in Table 2.

Age: LN patients were younger than both SLE-IgAN patients (*p* = 0.01) and primary IgAN patients (*p* < 0.01). There was no difference between SLE-IgAN vs. primary IgAN (*p* = 0.7).

Gender: difference was due to primary IgAN vs. other groups (*p* < 0.01). There was no difference between SLE-IgAN and SLE-LN (*p* = 0.4).

Proteinuria: primary IgA nephropathy differed significantly in proteinuria levels both from the SLE-IgAN and SLE-LN groups. (*p* < 0.001 for both). There was no difference between SLE-IgAN and SLE-LN groups (*p* = 0.367).

CYC: CYC usage differed significantly between the SLE-IgAN and SLE-LN groups (*p* = 0.047), while the other group comparisons did not show significant differences.

ACE-I: while a difference was seen between the groups, after applying the Bonferroni correction for multiple comparisons there were no statistically significant differences between the groups. The trend towards a lower use in the SLE-IgAN group warrants further investigation with a larger sample size for more definitive conclusions.

ESKD: this difference was primarily driven by the higher incidence of ESKD in the primary IgAN group compared to the SLE-LN group (*p* < 0.01).

Mortality: the primary driver of the difference was likely the higher mortality rate in the primary IgAN group compared to the SLE with lupus nephritis group; however, the difference was not statistically significant after adjusting for multiple comparisons.

## Discussion

To date, as many as 16 cases of SLE-IgAN have been reported [7–16]. Additionally, we present clinical features of two patients from our clinic and compare all these cases with the LN cohort from our clinic and one of the primary IgAN cohorts in the literature. The rate of end-stage renal disease in the group of SLE and IgAN was 11.1% and the mortality rate was 5.6%. In the LN cohort of 47 patients, the progression rate to end-stage renal failure was found to be 10.6% and the mortality rate was 10.6%.

The SLE is a chronic autoimmune disease, with approximately 50% of patients experiencing renal involvement during the disease course. While LN is a well-recognized manifestation, the existence of non-LN in SLE patients, such as IgA nephropathy, has been rarely reported. Hence, conducting a kidney biopsy in patients with renal abnormalities is crucial for an accurate diagnosis and treatment guidance [2]. Lupus nephritis is characterized by the accumulation of polyclonal immunoglobulins, predominantly of the IgG isotype and complement fractions (C1q, C3, C4) on the capillary basal membrane [1]. Conversely, in IgAN the IgA staining pattern should predominantly involve the mesangium, with or without additional staining of the capillary loops. While IgG and IgM may also be detected, their intensity should not surpass that of IgA, except in the case of IgM, which may be more prominent in sclerotic regions [17]. In the literature, some cases of atypical LN with mainly IgA deposition have been described. Although the coexistence of SLE and IgAN is not fully elucidated, it has been hypothesized that predominant mesangial IgA deposition may represent a distinct subtype of SLE nephritis; however, the absence of IgG and complement factors of the classical pathway (C1q and C4) does not align with typical LN [7, 11, 12].

The SLE predominantly affects women of childbearing age, with a female to male ratio of 9:1; however, male gender is associated with poorer renal outcomes. Lupus nephritis typically develops within 5 years of SLE diagnosis [18]. In contrast, IgAN primarily affects males during the second and third decades of life. In our study, the majority of SLE patients with IgAN were female, while there were more male patients in the primary IgAN group. The median age of patients with SLE and IgAN was 41.6 years, whereas the median age of patients in the primary IgAN cohort was 44 years. In the LN cohort, the median age of patients was 30 years. Similar to our study, Hongyan et al. described the clinical characteristics of a total of 5 SLE patients (2 males, 3 females) with predominant IgA deposition. They found some differences compared to primary IgAN, such as older age than primary IgAN, with an average of 42.2 ± 13.59 years [12].

The classical clinical presentation of LN typically includes persistent proteinuria, defined by the American College of Rheumatology classification criteria as 0.5 g per 24 h or a dipstick score of greater than 3+, or the presence of cellular casts or active urinary sediment. Elevated serum creatinine levels may also be indicative [1]. In contrast, primary IgAN is characterized by episodic gross hematuria or persistent microscopic hematuria, with concurrent proteinuria not always present. Approximately 40% of patients progress to end-stage renal disease, and serum complement levels are typically normal [17]. In our study, the vast majority of SLE patients with IgAN presented with varying levels of proteinuria and microscopic hematuria at initial presentation. Of the cases four presented with nephrotic range proteinuria, while two patients presented with acute kidney injury. Despite treatment, renal function continued to deteriorate in these cases, ultimately leading to end-stage renal disease.

In SLE patients, high titers of anti-dsDNA antibodies, often in conjunction with decreased levels of complement proteins C3 and C4, are typically associated with disease flare, particularly renal involvement. Conversely, serum complement levels are expected to be normal in cases of primary IgAN [19]. In our study, approximately half of the SLE with IgAN patients exhibited elevated anti-dsDNA antibodies and decreased complement levels, underscoring the importance of renal biopsy and the likelihood of non-LN in the context of serologically or clinically active lupus. In this context, low complement levels may reflect extrarenal lupus activity [11, 13].

The differentiation between lupus nephritis and IgAN is crucial due to differences in therapeutic management and prognostic outcomes. Treatment for LN typically involves immunosuppressive therapy tailored to the histopathological class of the disease, with antimalarial drugs recommended for all patients unless contraindicated [18]. In recent KDIGO (Kidney Disease Improving Global Outcomes) guidelines for IgAN (public review draft), the initial therapeutic strategy predominantly emphasizes the management of blood pressure and the reduction of proteinuria through the utilization of renin-angiotensin-aldosterone system (RAAS) inhibitors, as well as dual endothelin-angiotensin receptor antagonism (DEARA) and sodium-glucose cotransporter-2 inhibitors (SGLT2i), either as monotherapy or in combination. Notably, targeted-release budesonide (nefecon) is the only agent shown to reduce levels of pathogenic IgA and IgA immune complexes, and a 9-month course is recommended for patients at risk of progressive renal function decline. Systemic glucocorticoids are generally used sparingly in IgAN; however, if nefecon is unavailable, patients at higher risk of progressive renal deterioration may benefit from a short duration, reduced dose glucocorticoid regimen, alongside antimicrobial prophylaxis and guided by a thorough toxicity risk assessment [20].

In this study, the vast majority of SLE with IgAN patients showed renal function improvement after treatment with glucocorticoids, with or without immunosuppressive drugs. Hongyan et al. also demonstrated the beneficial effect of glucocorticoids and immunosuppressive treatment on SLE with IgAN [12]; however, a 19-year-old female patient diagnosed with cerebral lupus died due to an unknown systemic infection [7]. Another patient, a 72-year-old man presenting with acute progressive glomerulonephritis, received high-dose pulse glucocorticoid therapy and cyclophosphamide pulse treatment but failed to recover renal function, necessitating continued hemodialysis [15]. Similarly, Patel et al. reported on a 74-year-old woman with known SLE and chronic kidney disease attributed to diabetes mellitus and hypertension, who presented with acute kidney injury. Renal biopsy findings revealed IgAN with crescent formation. Despite treatment with pulse methylprednisolone and plasmapheresis, the renal function did not recover, leading to initiation of hemodialysis [14]. These cases underline that when IgAN is complicated by acute glomerulonephritis and crescent formation, patients may exhibit poor renal outcomes despite glucocorticoid and immunosuppressive drug therapy.

Lupus nephritis stands as a significant contributor to morbidity and mortality in SLE, with approximately 10% of cases progressing to end-stage renal disease [1]. In the literature, the reported mortality rate for proliferative LN (classes III, IV, or III/IV + V) is 5–25% and the progression rate to kidney failure is 10–30% [21]. In our LN cohort of 47 patients, the progression rate to end-stage renal failure was found to be 10.6% and the mortality rate was 10.6%. In SLE with IgAN group, the progression rate to kidney failure was found to be 11.1% and the mortality rate was 5.6%. On the other hand, in the primary IgAN group, 34% of 215 patients progressed to end-stage kidney disease, with a reported mortality rate of 20%, primarily due to cardiovascular diseases [6]. These findings may suggest that SLE with IgAN may exhibit slightly better renal outcomes compared to primary IgAN.

The main limitation of our study is that the data were compiled from case reports and case-based reviews in the literature, highlighting the need for long-term follow-up data on these cases; however, our study stands out as a more comprehensive comparison of the clinical characteristics of patients with SLE and IgAN with the primary IgAN population.

The reason why certain SLE patients are more susceptible to developing IgAN remains unclear. The shared genetic and environmental backgrounds of SLE and IgAN support our hypothesis that these two diseases may coexist due to similar immunological mechanisms.

In summary, SLE with IgAN exhibits some distinct clinical characteristics from primary IgAN. As evidenced by the follow-up of reported cases in the literature, SLE with mainly IgA deposition may present a more indolent course compared with primary IgAN; however, when IgAN is complicated by acute glomerulonephritis and crescent formation, such patients may demonstrate poor renal outcomes despite treatment with glucocorticoids and immunosuppressive drugs. Hence, it is imperative to perform a kidney biopsy in lupus patients with renal abnormalities, as the diagnosis may not always be LN and it should be kept in mind that IgAN may also coexist in lupus patients. An accurate diagnosis is crucial for guiding appropriate therapeutic regimens.

Nevertheless, more data are needed on clinical features, treatment approaches, and prognostic outcomes in patients with SLE and IgAN. Therefore, prospective and comparative studies with larger patient populations should be conducted in the future.

## Data Availability

The datasets used and analyzed during the current study are available from the corresponding author on reasonable request
